# Construction and validation of a RARRES3-based prognostic signature related to the specific immune microenvironment of pancreatic cancer

**DOI:** 10.3389/fonc.2024.1246308

**Published:** 2024-02-05

**Authors:** Yimeng Sun, Xiaoyan Wang, Lin Yao, Rong He, Changfeng Man, Yu Fan

**Affiliations:** ^1^ Cancer Institute, Affiliated People’s Hospital of Jiangsu University, Zhenjiang, Jiangsu, China; ^2^ Department of Gastroenterology, Suqian First People’s Hospital, Suqian, Jiangsu, China

**Keywords:** tumor immune microenvironment, RARRES3, immunotherapy, pancreatic cancer, prognostic signature

## Abstract

**Background:**

Tumor immune microenvironment (TiME) is prognostically instructive in Pancreatic adenocarcinoma (PAAD). However, the potential value of TiME-related genes in the individualized immunotherapy of PAAD has not been clarified.

**Methods:**

Correlation between Immune-Related Genes (IRGs) and immune-related transcription factors (TFs) was performed to prove the immune correlation of selected genes. Immune-related molecular subtypes were identified by consensus clustering. The TiME-score, an immune microenvironment-related prognostic signature for PAAD, was constructed using minimum absolute contraction and selection operator regression (Lasso-Cox). The International Cancer Genome Consortium (ICGC) dataset validated the reliability of TiME-score as external validation. Single-cell samples from GSE197177 confirmed microenvironment differences of TiME-score hub genes between tumor and its paracancer tissues. Then, RARRES3, a hub gene in TiME-score, was further analyzed about its upstream TP53 mutation and the specific immune landscape of itself in transcriptome and Single-cell level. Eventually, TiME-score were validated in different therapeutic cohorts of PAAD mice models.

**Results:**

A 14-genes PAAD immune-related risk signature, TiME-score, was constructed based on IRGs. The differences of TiME-score hub genes in single-cell samples of PAAD cancer tissues and adjacent tissues were consistent with the transcriptome. Single-cell samples of cancer tissues showed more pronounced immune cell infiltration. The upstream mutation factor TP53 of RARRES3 was significantly enriched in immune-related biological processes. High RARRES3 expression was correlated with a worse prognosis and high macrophages M1 infiltration. Additionally, the immunohistochemistry of hub genes AGT, DEFB1, GH1, IL20RB, and TRAF3 in different treatment cohorts of mice PAAD models were consistent with the predicted results. The combination of immunotherapy, chemotherapy and targeted therapy has shown significantly better therapeutic effects than single drug therapy in PAAD.

**Conclusion:**

TiME-score, as a prognostic signature related to PAAD-specific immune microenvironment constructed based on RARRES3, has predictive value for prognosis and the potential to guide individualized immunotherapy for PAAD patients.

## 1 Introduction

PAAD has been known for its high morbidity rate of 7.7 age-standardized rate, high mortality rate of 4.9 age-standardized rate, and low 5-year survival rate of 4.3%. Its morbidity and mortality rates are increasing year by year (1, 2). Due to various factors such as non-specific symptoms, difficulty in a tissue biopsy, lack of good screening methods, rapid tumor progression, and low response rate to treatment, approximately 80% of patients are already advanced at the time of diagnosis, with a median survival of only 7 months (3). Currently, immunotherapy modalities such as immune checkpoint inhibitors, adoptive cell transfer therapy, and molecularly targeted drugs are playing an increasingly important role as emerging therapeutic trends (4). However, the immunotherapeutic results of PAAD were still not satisfactory.

TiME is a self-serving immune tissue environment created by cancer for its growth. Tumor-produced cytokines and chemokines, oncogenes, and mutational landscapes influence the composition of TiME (5). The heterogeneity of TiME partly depends on the adaptability of infiltration and the diversity and plasticity of innate immune cells (6). Additionally, Cancer-associated fibroblasts that crosstalk with immune cells also form immune highly suppressed TiME by secreting various carrier molecules that allow cancer cells to evade immune system surveillance (7). Therefore, understanding the complex role of different components of TiME in tumor progression, reversing this highly immunosuppressive TiME, and thus developing more individualized immunotherapy regimens based on TiME differences in different patients has become a new research direction in PAAD treatment in recent years. Besides, mutated or dysregulated immune-associated TFs mediate the abnormal expression of IRGs, which in turn block the cell differentiation and cell death gene expression programs (8). Aberrant immune-associated TFs represent a unique class of drug targets. In practical therapeutics, drug development can be pursued by targeting TFs. Gene mutation is another vital factor in the progression of PAAD. Mutations of oncogene TP53 can allow the transfer of its downstream proteins to neighboring cancer cells and macrophages via the extracellular vesicle detached from cancer cells, regulating their release of tumor-supporting cytokines and thus leading to TiME reprogramming (9). Although the impact of TiME is complex, most of the current studies evaluating the effect of TiME on PAAD have focused on the construction of models for a few immune cells or tumor differential genes. Hence, there is an urgent need for a novel and reliable prediction method that combines immune-related TFs and genomic changes to evaluate TiME and guide individualized immunotherapy for PAAD patients.

In this study, we identified 14 key IRGs with the best prognostic value, and construct a risk prediction signature named TiME-score. The TiME-score was validated in the ICGC cohort, Immunohistochemistry and Single-cell samples. Then, the TiME landscape of the hub gene RARRES3 and its upstream mutation factor TP53 were investigated. Clinical prognostic analysis was also performed for RARRES3. Additionally, immune cell infiltration, immune check sites, and immune escape were analyzed in the high and low risk groups of TiME-score. Finally, the hub genes were validated in different drug administration cohorts of PAAD mice models. The value of immune, chemical and targeted combination therapy in PAAD has also been proved. This study envisions exploring prognostic indicators to help oncologists determine individualized PAAD treatment strategies.

## 2 Materials and methods

### 2.1 Data acquisition and processing

**Figure 1:**
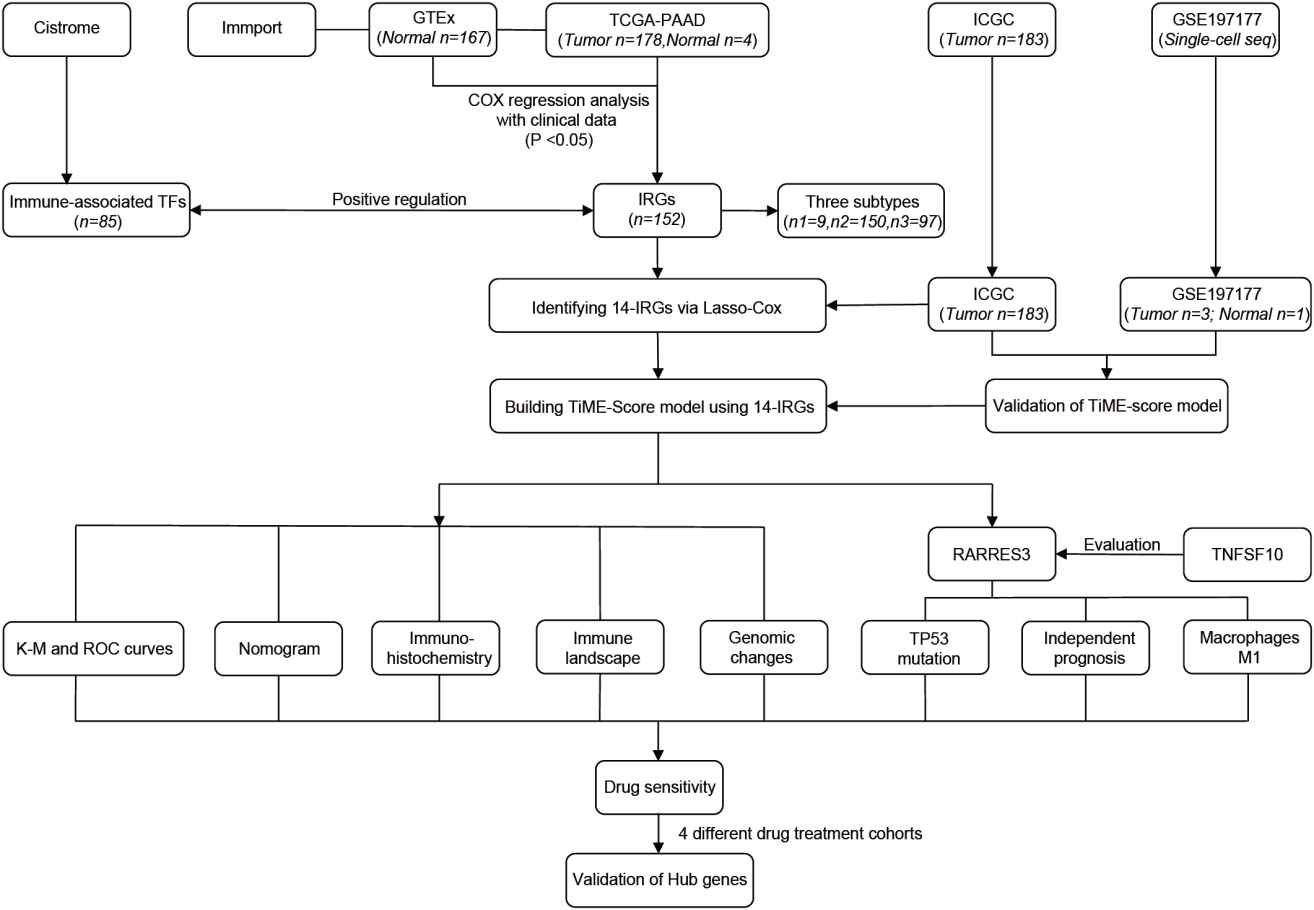
The flowchart of our study process.

The general process of this study is shown below (**Figure 1**). First, 178 PAAD samples and 4 normal tissue samples were downloaded and collected from the The Cancer Genome Atlas (TCGA, http://portal.gdc.cancer.gov) database to obtain their quantitative gene expression data and clinical data. The TCGA-PAAD cohort was used as a training cohort. Transcriptome data from 167 normal pancreatic tissues were obtained from the Genotype-Tissue Expression (GTEx, https://gtexportal.org/) database. Matching TCGA with the data from GTEx. Next, 183 PAAD samples were downloaded from the ICGC (https://icgc.org/) database as an external validation cohort. The dataset of IRGs was obtained from the Immport (https://www.immport.org) database. The dataset of immune-related TFs was obtained from the Cistrome (https://cistrome.org) database. In addition, mutation data of PAAD and maf files of mutect versions of somatic mutations were downloaded from the TCGA database and used to analyze TP53 mutations and evaluate TMB scores. Single cell sequencing samples of 3 PAAD tissues and 1 adjacent normal tissue were obtained from GSE197177 of the Gene Expression Omnibus database (GEO, https://www.ncbi.nlm.nih.gov/geo/).

### 2.2 Screening of IRGs and TFs and their interactions

The differentially expressed genes were screened by the Wilcox test using R software on the obtained data. The pheatmap package was used to draw volcano and heat maps of the differentially expressed genes. Among the screened differentially expressed genes, differentially expressed IRGs were identified using the above method. Screening criteria were false discovery rate (FDR) < 0.05 and | log2 fold-change (FC)| > 1. COX regression analysis was performed on the differential IRGs and clinical prognostic information, which in turn filtered out prognostically relevant IRGs. In addition, 318 immune-related TFs were downloaded from Cistrome and screened for differentially expressed TFs using the same method as above. The selection criteria were the same as for the IRGs. Correlation coefficients > 0.4 and p < 0.0001 were used as thresholds for correlation analysis between differentially expressed TFs and IRGs. Cytoscape software was used to map their interaction regulatory networks.

### 2.3 Unsupervised clustering and identification of molecular subtypes

The limma package was used to perform unsupervised clustering analysis on 178 PAAD patients from TCGA to identify different immune-related molecular subtypes. The ConsensusClusterPlus package was used to perform a consensus clustering algorithm with 1000 iterations to ensure classification stability. The survivor and survminer packages were used to plot Kaplan-Meier (K-M) survival curves. The estimate package was used to evaluate StromalScore, ImmuneScore, and ESTIMATEScore for 3 subtypes.

### 2.4 Construction and validation of TiME-score

Discovering differentially expressed genes (DEGs) with significant prognostic value were identified using univariate Cox regression analysis. LASSO-Cox regression analysis was performed using the glmnet package to further narrow down the candidate genes (10). The prognostically relevant IRGs were selected and the TiME-score was calculated for PAAD patients (β: coefficients, Exp: gene expression level):


$$\text{TiMEscore}=\sum (\beta_{i}\times {\text{Exp}}_{i})$$

The TCGA-PAAD cohort was divided into high-risk and low-risk subgroups based on the median TiME-score. K-M curves and receiver operating characteristic (ROC) curves were used to assess the accuracy and specificity of the TiME-score. Moreover, the ICGC dataset was used as an external validation set to verify the predictive value of the signature.

### 2.5 Independent prognostic analysis of TiME-score

To determine whether the TiME-score could be used as an independent predictor of prognosis, independent prognostic analyses were performed using univariate and multifactorial Cox. Patients with PAAD were divided into groups based on clinical information such as age (≤65 or >65 years), gender, pathological grade, clinical stage, T (T1-2 or T3-4), N (N0 or N1), and M (M0 or M1). On this basis, the TiME-scores of the patients were compared between groups. The beeswarm package was used to assess the correlation between prognostic characteristics and the above clinical parameters. Additionally, a nomogram was created using the rms package to accurately predict the 1-year, 2-year, and 3-year overall survival (OS) of PAAD patients. Also, the predictive performance of the nomogram was evaluated using calibration curves.

### 2.6 Expression, immunohistochemical and proteomic differences of hub genes in TiME-score

The ggplot2 package was used to analyze the differential expression of the hub genes in the TiME-score among the TCGA-PAAD cohort and the GTEx cohort. The Human Protein Atlas (HPA, https://www.proteinatlas.org/) database (11) is a database that provides extensive transcriptomic and proteomic data for specific human tissues and cells. Samples used for immunohistochemistry by the HPA do not correspond to samples in the TCGA dataset. All tissues were collected from the Uppsala Biobank and RNA samples were extracted from frozen tissue sections. The differences in protein expression of hub genes between PAAD and normal pancreatic tissues were validated using Immunohistochemistry in HPA. The Clinical Proteomic Tumor Analysis Consortium database (CPTAC, https://proteomics.cancer.gov/programs/cptac) is a research project designed to accelerate understanding of cancer through the application of large-scale proteomic and genomic analysis or proteomics. The University of ALabama at Birmingham CANcer data analysis Portal (UALCAN, https://ualcan.path.uab.edu/index.html) was used to analyze protein expression in the CPTAC database.

### 2.7 Differential expression and distribution of hub genes in TiME-score at single-cell level

Single-cell RNA sequencing count matrices for all 3 PAAD tissues (GSM5910784, GSM5910787 and GSM5910789) were downloaded from GSE197177. The 3 matrices were combined into a single Seurat object using the CreateSeuratObject function (Seurat R package, version 4.3.0). Cells with >2500 or <200 genes detected in a single cell, or with UMI counts >5% from mitochondrial sources are considered low quality cells. These low-quality cells were filtered out and single-cell data were normalized. Next, the FindNeighbors function was used to identify similar cells. The FindClusters function (resolution = 0.4) was used to identify major cell clusters. Based on this, DimPlot was used to visualize the UMAP non-linear dimensionality reduction results of these cell clusters. Cell clusters were identified using the “SingleR” package and annotated based on marker genes obtained from previous studies. The accuracy of the annotation was verified using the plotScoreHeatmap function. The number and distribution of target gene expressions were marked on UMAP using the FeaturePlot function. In addition, the same analysis described above was done on a single cell sample (GSM5910786) from 1 PAAD adjacent to normal tissue in GSE197177, which served as a control.

### 2.8 Correlation analysis between TP53 mutations and immune landscape

An overview graph of TP53 mutations in PAAD was mapped using the maftools package. Using gene set enrichment analysis (GSEA) software (version: v4.2.3; www.gsea-msigdb.org/gsea/downloads.jsp) (12), determine immune-related biological process differences between TP53 samples from the TCGA-PAAD cohort (wild group n=62, mutant group n=81). An annotated gene set file (c5.bp.v7.1.symbols.gmt) was selected as the reference gene set (p<0.05). The relative proportion of all 22 tumor-infiltrating immune cells (TICs) in each sample was estimated using the CIBERSORT package (number of cycles = 1000) (13). This algorithm was run more than three times with identical results. Subsequently, the infiltration of immune cells and the correlation between immune cells in the wild and mutant groups were analyzed sequentially.

### 2.9 Exploring the prognostic value of RARRES3

Considering that both RARRES3 and TNFSF10, the hub genes of TiME-score, are downstream genes of TP53. K-M curves and ROC curves of RARRES3 high and low expression groups were plotted with survival and survminer packages. The effects of age, gender, grade, stage, and RARRES3 expression on independent prognosis were assessed by univariate and multifactorial Cox analysis. The same method was used to analyze TNFSF10 as control and validation. In addition, the correlation between RARRES3 and clinical indicators was evaluated.

### 2.10 Immunoscape analysis of RARRES3

Enrichment analysis of RARRES3 was performed using the clusterProfiler package and the GSEA enrichment results of RARRES3 were plotted using the enrichplot package. This was used to assess the abundance of different immune-related functions or pathways associated with RARRES3 expression. Next, the abundance of all 22 TICs was estimated using the CIBERSORT algorithm, which in turn ranked the correlation with RARRES3 expression for each immune cell. Moreover, the correlation between RARRES3 and immune checkpoints was analyzed by the corrplot package. TNFSF10 analysis was applied in the same way, as control and validation.

### 2.11 Evaluation of immune landscapes, gene mutations, and immunotherapy responses in high- and low-risk groups

The enrichplot package was used for Gene Ontology (GO) and Kyoto Encyclopedia of Genes and Genomes (KEGG) enrichment analysis of the high-risk and low-risk groups. 22 TICs in each sample were estimated using the CIBERSORT package. Mutations and TMB scores were plotted for each sample using the maftools package. The correlation between TiME-score and TMB was plotted. This study also explored the association between TiME-score and the expression of key genes of immune checkpoint blockade. Also, the Tumor Immune Dysfunction and Exclusion (TIDE) algorithm was used to evaluate the potential response of PAAD patients in different risk score groups to immunotherapy.

### 2.12 Validation of hub genes in immune-, targeted- and chemo-therapy mice PAAD model

Half-maximal inhibitory concentrations (IC50) were estimated by the pRRophetic package. The filtering condition was P< 0.05. Integrating the results of drug sensitivity analysis and the common clinical drugs for PAAD, the hub genes of TiME-score were validated in the immune-, targeted- and chemo-therapy mice PAAD model in this study. First of all, 6-8 weeks female mice (C57BL/6), mice pancreatic cancer PANC-02 cells, Nanoparticle Albumin-Bound Paclitaxel (Abraxane, Jiangsu Hengrui Pharmaceutical Co., Ltd., Specification: 100mg, Lot No. 220310AF), gemcitabine, recombinant human vascular endothelial Inhibin Injection (Endostar, Shandong Xiangsheng Biopharmaceutical Co., Ltd., Specification: 15mg/3ml, Lot No.: 202109053), and PD1 monoclonal antibody (Jiangsu Hengrui Pharmaceutical Co., Ltd., Specification: 200mg, Lot No.: 202009043A) were prepared. Then, the cultured PAAD cell suspension (concentration: 5×107 cells/ml) of PANC-02 mice was collected and injected subcutaneously in the right axilla of mice with 0.1 ml each. The diameter of the transplanted tumor was observed, and the mice were randomly divided into 4 groups of 6 mice each when the tumor grew up to 100 mm³. Simultaneously, 4 groups of mice were started to be administered separately according to the dosing regimen (**Supplementary Table 1**). 15 days later, immunohistochemical verification of hub genes AGT, DEFB1, GH1, IL20RB, and TRAF3 was performed in PAAD tissues of mice in each group. **Supplementary Table 2** presents information about the antibodies used for immunohistochemistry.

### 2.13 Statistical analysis

All data analyses were based on R software (version 4.1.3). The Wilcoxon test was used to compare the differences in gene expression levels between PAAD and normal tissues. The Spearman coefficient test was used to perform correlation tests. Image-Pro Plus (version 6.0) and GraphPad Prism software (version 8.0.2) were used for immunohistochemistry analysis. The threshold of statistical significance was considered to be P < 0.05 if not explicitly mentioned.

## 3 Results

### 3.1 IRGs selection and positive correlation with immune-related TFs

**Figure 2:**
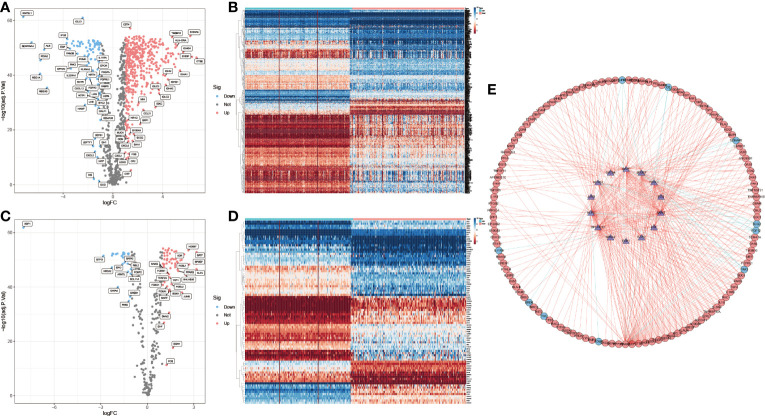
IRGs selection and positive correlation with immune-related TFs. **(A, B)** Up-regulated (red, n=438) and down-regulated (blue, n=88) differentially expressed IRGs. **(C, D)** Up-regulated (red, n=58) and down-regulated (blue, n=27) immune-related differentially expressed TFs. **(E)** Interaction of prognosis-related IRGs with TFs. (outer circles: IRGs; inner circle: TFs).

By integrating the PAAD data of TCGA and GTEx (Normal: n=171, Tumor: n=178), 576 differentially expressed IRGs were selected (**Figures 2A, B**). Using the same method, 85 differentially expressed immune-associated TFs were obtained (**Figures 2C, D**). A total of 152 prognosis-related IRGs were obtained by COX regression analysis of PAAD immune-related differential genes and clinical prognosis data (P < 0.05), where Hazard ratio (HR) > 1 were prognostic risk factors and 0 < HR < 1 were prognostic protective factors (**Supplementary Table 3**). Subsequently, the interaction network between prognosis-related IRGs and TFs was constructed, and most of them were found to be positively regulated (**Figure 2E**). This indicates that the immunological relevance of the 152 IRGs screened in PAAD is reliable.

### 3.2 Identification of immune-related molecular subtypes

**Figure 3:**
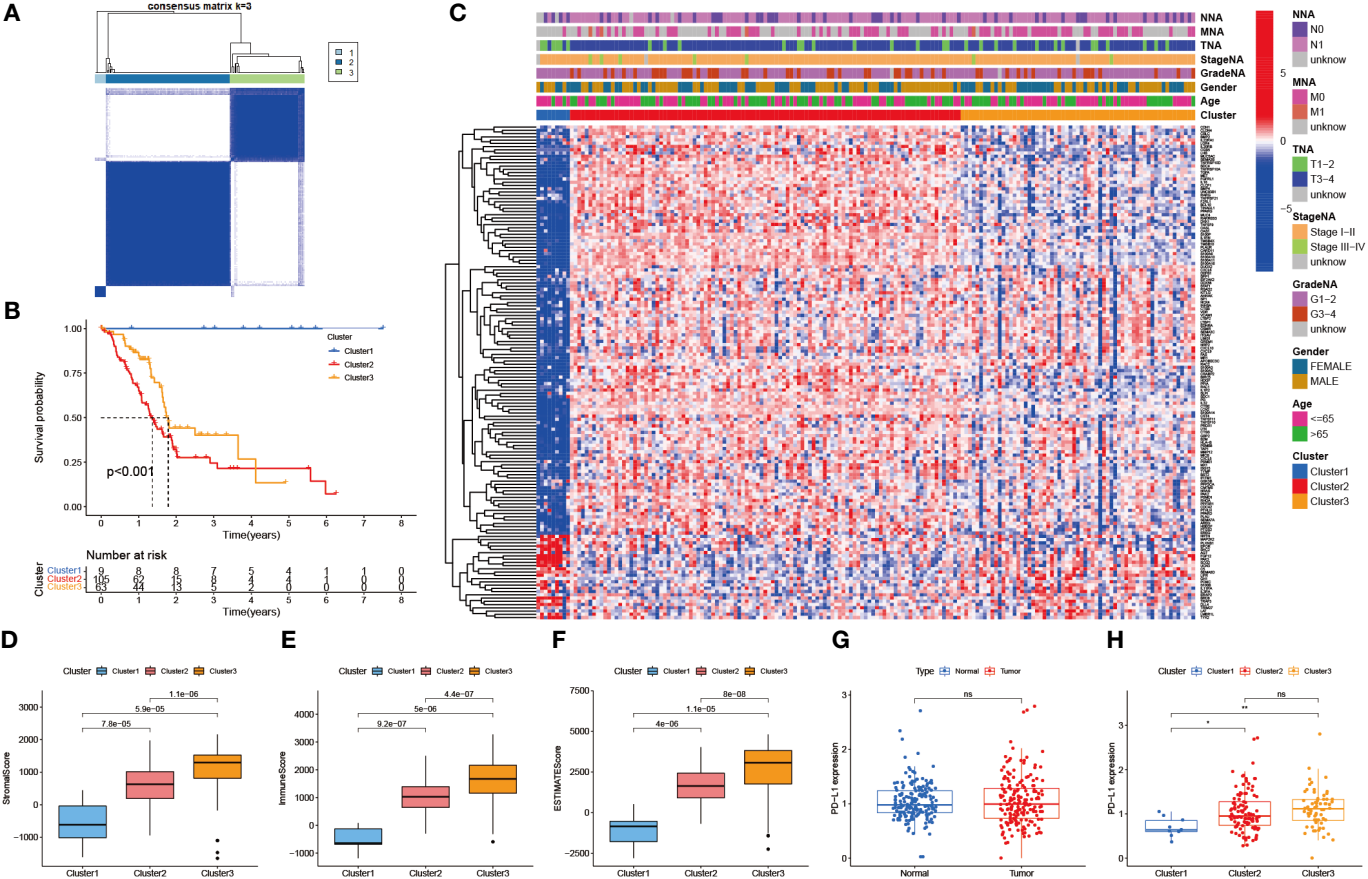
Identification of immune-related molecular subtypes. **(A)** 3 molecular subtypes of the TCGA-PAAD cohort. (cluster1: n=9, cluster2: n=105, cluster3: n=63) **(B)** Survival analysis of the 3 subtypes. (p < 0.001) **(C)** Heat map of the correlation between the 3 subtypes and clinical risk factors. **(D-F)** TiME evaluation of the 3 subtypes. (StromalScore, ImmuneScore, and ESTIMATEScore) **(G)** PD-L1 expression differences between normal and PAAD cohorts. **(H)** PD-L1 expression differences between the 3 subtypes.

Consensus cluster analysis was performed on all PAAD samples with survival information in the TCGA-PAAD cohort. Intra-group correlations were highest and inter-group correlations were lowest when the clustering variable k = 3 (**Figure 3A**, **Supplementary Figure 1**). K-M survival analysis showed that cluster 1 had a much higher survival advantage than clusters 2 and 3, while cluster 3 had a slightly higher survival advantage than cluster 2 (P<0.001, **Figure 3B**). Combined with the clinical indicators, it was found that the better prognosis of cluster 1 mainly corresponded to lower grade and stage, while the worse prognosis of clusters 2 and 3 mainly matched higher grade and stage (**Figure 3C**). Then, StromalScore, ImmuneScore, and ESTIMATEScore were applied to TiME for each of the three clusters (**Figures 3D-F**). The results showed that all three scoring groups of clusters 1, 2, and 3 showed a progressive relationship. Besides, the difference in programmed death-ligand 1 (PD-L1) expression between the normal and PAAD groups was not statistically significant, but there was a significant progressive trend of PD-L1 expression levels in clusters 1, 2, and 3 (**Figures 3G, H**). It indicates that this consensus clustering can better assess immune escape and guide immunotherapy than conventional classification. Interestingly, although clusters 1,2, and 3 were progressive in immune cell infiltration analysis and immune escape prediction, the survival analysis results of clusters 2 and 3 were reversed in the K-M curves. We speculate that this may be related to insufficient sample size.

### 3.3 TiME-score construction and validation

**Figure 4:**
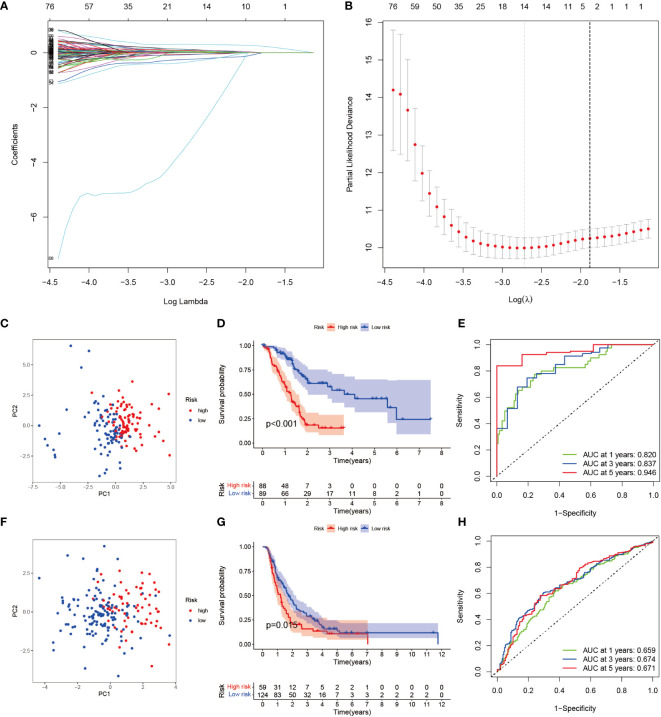
TiME-score construction and validation. **(A)** Disclosure of partial likelihood bias. **(B)** LASSO-COX regression method to identify 14 PAAD immune-related model genes. **(C)** Principal component PCA analysis of different risk groups in the TCGA-PAAD cohort. **(D)** Survival analysis of patients with high and low risk groups in the TCGA-PAAD cohort. **(E)** ROC curves for the TCGA-PAAD cohort at 1, 3, and 5 years. **(F-H)** The same approach to analyze the ICGC cohort.

To seek a better assessment method for predicting PAAD immune-related prognosis, we constructed a signature based on 152 PAAD prognosis-related IRGs. The TCGA-PAAD cohort was used as the training set and the ICGC cohort as the validation set. By using LASSO-COX regression analysis, 14 hub genes with the best prognostic value were finally identified: AGT, CXCL9, DEFB1, ERAP2, GH1, IL1R2, IL20RB, LMBR1L, MET, PLAU, RARRES3, TNFSF10, TRAF3, and TYK2. An immune-prognostic signature, TiME-score, for PAAD was constructed (**Figures 4A, B**, **Table 1**). Patients from the TCGA-PAAD cohort were divided into a high-risk group (n=88) and a low-risk group (n=89) according to the median risk cutoff. Principal component analysis of the different risk groups showed satisfactory separation (**Figure 4C**). In addition, K-M survival curves showed significant differences in survival time between patients in the high-risk and low-risk groups (**Figure 4D**). Meanwhile, the area under the curve (AUC) of the ROC curve at 1, 3, and 5 years was 0.820, 0.837, and 0.946 (**Figure 4E**). This demonstrated the promising sensitivity and specificity of the TiME-score. As a validation set, the results of the analysis of the ICGC cohort further confirmed the reliability of the TiME-score (**Figures 4F-H**).

**Table 1:** Hub genes and their correlation coefficients of TiME-score.

Gene	Coef
AGT	-0.0660957654315
CXCL9	0.0565979901850
DEFB1	0.0159141220456
ERAP2	0.1183259886127
GH1	-3.2585152871784
IL1R2	0.0754363636720
IL20RB	0.0479147299316
LMBR1L	-0.0542551297667
MET	0.3099034174497
PLAU	0.0060506575729
RARRES3	0.0922729854407
TNFSF10	0.1101191503172
TRAF3	-0.3324332060335
TYK2	-0.2402690980103

### 3.4 Independent prognostic value of TiME-score

**Figure 5:**
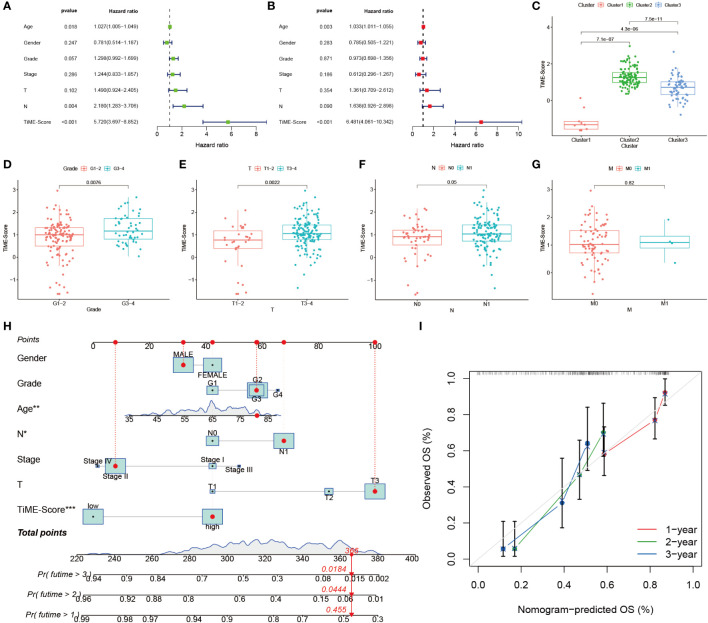
Independent prognostic value of TiME-score. **(A)** Univariate COX regression analysis. **(B)** Multivariate COX regression analysis. **(C)** TiME-score differences among 3 subtypes. **(D-G)** TiME-score differences among different pathological grades, T, N, and M. **(H)** Nomogram predicting 1, 2, and 3-year OS of PAAD patients. (∗P < 0.05; **P < 0.01; ∗∗∗ P < 0.001) **(I)** Calibration curve of this Nomogram.

To investigate whether the prognostic value of the TiME-score was independent of other clinical factors, we conducted univariate and multifactorial COX regression analyses on TiME-score (**Figures 5A, B**). The results showed that TiME-score remained an independent prognostic factor after adjusting for relevant clinical characteristics (P<0.001). Then, the comparison of PAAD patients with different clinical indicators revealed that TiME-score was consistent with clinical malignancy, in consensus cluster grouping (P<0.001), Grade grouping (P=0.0076), T grouping (P=0.0022), and N grouping (P=0.05) (**Figures 5C-G**). Notably, the trend in the 3 clusters of consensus clustering (**Figure 5C**) was following the results of survival analysis (**Figure 3B**), which reflects the superior predictive capability of the TiME-score. Besides, to define an individualized scoring system for each patient, a nomogram combining age, gender, clinical grade, pathological stage, and TiME-score was created to predict the 1-, 2-, and 3-year survival of PAAD patients (**Figure 5H**). Specifically, the 1-, 2-, and 3-year survival rates for a given patient can be calculated from this nomogram we constructed. The points corresponding to the patient’s TiME-score, gender, age, pathological grades, and clinical stages are summed. The resulting total points correspond to the “Total points” axis at the bottom of this nomogram. A vertical line is drawn through the point. The intersection of this vertical line with the three different survival axes, Pr(futime>1), Pr(futime>2), and Pr(futime>3), corresponds to the survival rate of the patient at the three time points of 1 year, 2 years, and 3 years, respectively. The calibration curve showed that the predicted probability values of OS at 1, 2, and 3 years were similar to the actual, demonstrating the clinical value of this nomogram (**Figure 5I**).

### 3.5 Differential expression of TiME-score hub genes in transcriptome, immunohistochemistry and proteomics

**Figure 6:**
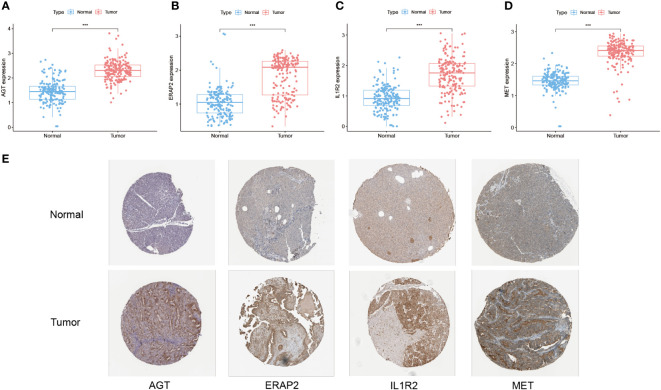
Differential expression of TiME-score hub genes in transcriptome, immunohistochemistry and proteomics. **(A–D)** Expression pattern of AGT, ERAP2, IL1R2, and MET in normal and PAAD samples from the PAAD and GTEx databases (“***”: P<0.001). **(E)** Immunohistochemistry of AGT, ERAP2, IL1R2, and MET in normal and PAAD samples from the HPA database.

Combining TCGA-PAAD and GTEx datasets, all 14 hub genes of TiME-score were analyzed for differential expression levels in tumor tissues and their adjacent normal pancreatic tissue samples (**Figures 6A-D**, **Supplementary Figure 2**). Subsequently, the immunohistochemical results of normal and PAAD samples for each hub gene under the same antibody treatment were selected from the HLA database and analyzed for comparison (**Figure 6E**). The results showed that there were 4 genes whose immunohistochemical results were consistent with the differential analysis of transcriptome. Specifically, their immunohistochemical results in normal tissues adjacent to the cancer were AGT (Staining: Not detected, Intensity: Negative, Quantity: None), ERAP2 (Staining: Not detected, Intensity: Negative, Quantity: None), IL1R2 (Staining: Low, Intensity: Weak, Quantity: 75%-25%), and MET (Staining: Low, Intensity: Weak, Quantity: >75%). In contrast, their immunohistochemical results in PAAD tissues were AGT (Staining: Medium, Intensity: Moderate, Quantity: 75%-25%), ERAP2 (Staining: Medium, Intensity: Moderate, Quantity: > 75%), IL1R2 (Staining: High, Intensity: Strong, Quantity: 75%-25%), MET (Staining: Medium, Intensity: Moderate, Quantity: >75%). In addition, GH1 (**Supplementary Figure 2C**), PLAU (**Supplementary Figure 2F**), and TNFSF10 (**Supplementary Figure 2H**) were not significantly different immunohistochemically, although they were statistically significant in terms of expression differences. Immunohistochemical results for TRAF3 (**Supplementary Figure 2I**) and TYK2 (**Supplementary Figure 2J**) showed opposite trends to the transcriptome intergroup differences. Immunohistochemical results for CXCL9 (**Supplementary Figure 2A**), DEFB1 (**Supplementary Figure 2B**), IL20RB (**Supplementary Figure 2D**), LMBR1L (**Supplementary Figure 2E**) and RARRES3 (**Supplementary Figure 2G**) were not retrieved and need further validation. We then analyzed the proteomics of all 14 hub genes of TiME-score in the CPTAC database using UALCAN. The results showed that the differences of protein expression between normal and PAAD cells of AGT (**Supplementary Figure 3A**), ERAP2 (**Supplementary Figure 3C**), MET (**Supplementary Figure 3D**), PLAU (**Supplementary Figure 3E**), TNFSF10 (**Supplementary Figure 3F**), and TRAF3 (**Supplementary Figure 3G**) consistent with transcriptome analysis (**Supplementary Figure 3I**). DEFB1 (**Supplementary Figure 3B**) and TYK2 (**Supplementary Figure 3H**) protein expression did not match the intergroup differences between cancer and adjacent normal tissues in the transcriptome.

### 3.6 Differential expression and distribution of hub genes in TiME-score at single-cell level

**Figure 7:**
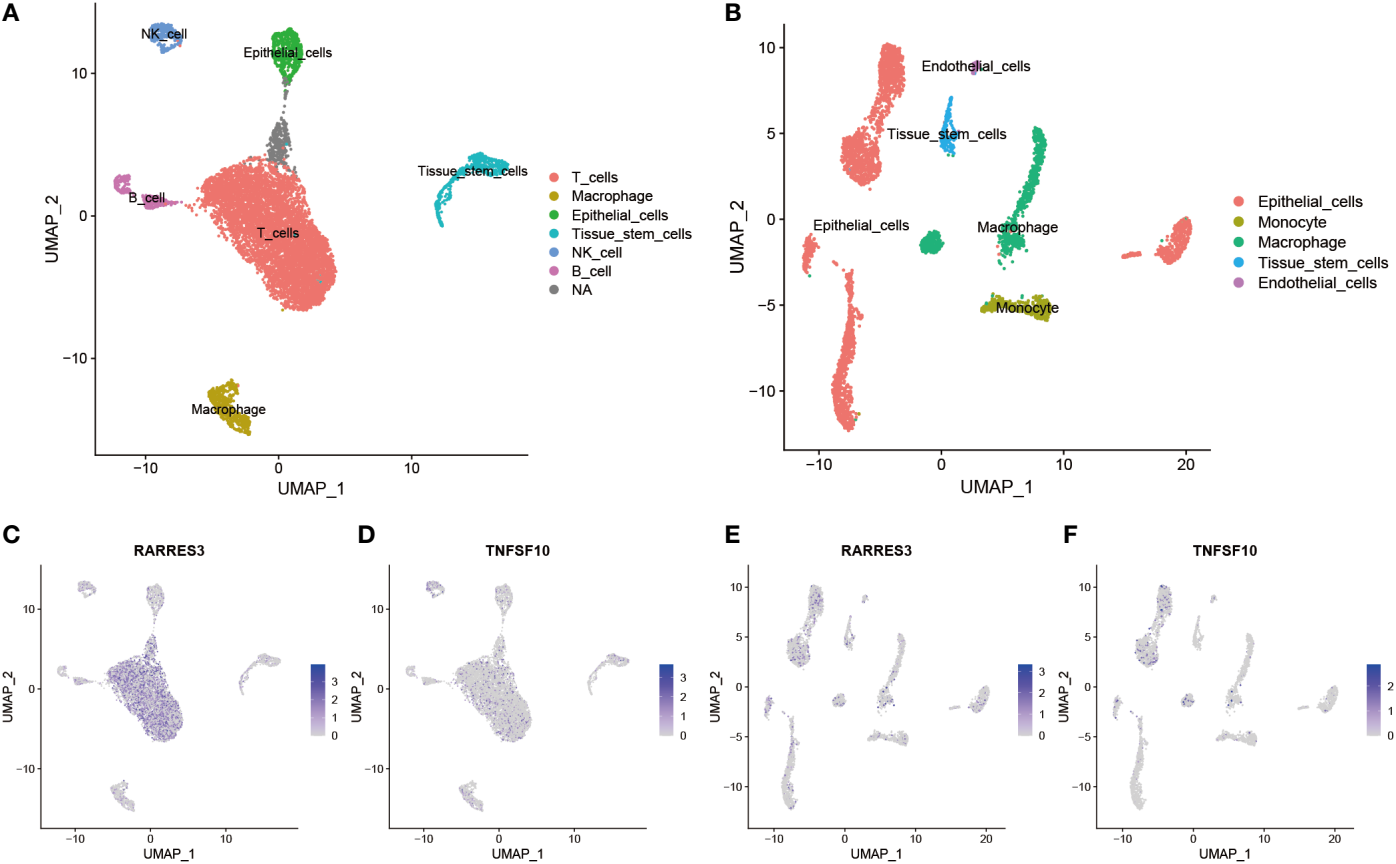
Differential expression and distribution of hub genes in TiME-score at single-cell level. **(A)** All cells in 3 PAAD single-cell samples were annotated as 6 different cell clusters. **(B)** All cells in 1 single-cell sample of normal tissue adjacent to cancer was annotated as 5 different cell clusters. **(C-F)** Distribution and expression differences of RARRES3 and TNFSF10 in each single cell clusters between cancerous tissues **(C, D)** and normal tissues adjacent to cancer **(E, F)**.

To further explore the expression differences of all 14 TiME-score hub genes between PAAD tissues and their adjacent normal tissues. A comparative analysis of 3 PAAD single-cell samples and 1 para-cancer sample from GSE197177 were performed. The results showed that cancer tissues were clearly separated into 10 cell clusters (**Supplementary Figure 4A**), and these cell clusters were annotated into 6 different cell types (**Figure 7A**), which were T cells, Macrophages, Epithelial cells, Tissue stem cells, NK cells, and B cells. The normal tissue was clearly separated into 13 cell clusters (**Supplementary Figure 5A**), and these cell clusters were annotated into 5 different cell populations (**Figure 7B**), named Epithelial cells, Monocytes, Macrophages, Tissue stem cells, and Endothelial cells. The accuracy of annotation was verified one by one (**Supplementary Figures 4B**, **5B**). Overall, the infiltration levels of T cells, NK cells, and B cells, which are immune infiltrating cells in cancer tissues, were significantly higher than those in normal tissue samples adjacent to cancer. This suggests that the development of PAAD is accompanied by more pronounced immune cell infiltration. Furthermore, a comparison of all 14 hub genes expression counts data in single-cells of pancreatic cancer (**Supplementary Figure 4C**) and its para-cancerous tissues (**Supplementary Figure 5C**) revealed that the expression levels of RARRES3 and TNFSF10 were most significantly increased in PAAD single-cell samples compared with normal. In contrast, the expression level of DEFB1 was most significantly decreased in PAAD single-cell samples compared with normal tissues. They were consistent with the results of transcriptome analysis. Interestingly, GH1 gene expression was not detected in any cell clusters of normal tissue. This suggested that although the overall expression level of GH1 was low in both PAAD and para-cancerous tissues, the expression level of GH1 in cancerous tissues was still significantly higher than that in para-cancerous tissues, which was different from the results of our transcriptome analysis, so we verified the reliability of GH1 in subsequent animal experiments. Besides, the expression differences of the remaining 10 genes, AGT, CXCL9, ERAP2, IL1R2, IL20RB, LMBR1L, MET, PLAU, TRAF3, and TYK2, were consistent with the trend of inter-group differences in transcriptome. Subsequently, consider the value of most significantly expression in single-cell samples of PAAD cancer tissues, we labeled the expression levels and expression distribution of RARRES3 and TNFSF10 in the clustered UMAP cell cluster descending plots of cancer and para-cancer tissues. Combined with the violin plots of expression distribution, we found that RARRES3 was mainly expressed in T cells, B cells and Tissue stem cells clusters of cancer tissues (**Figure 7C**) and Epithelial cells clusters of normal tissues (**Figure 7D**). TNFSF10 was mainly expressed in Tissue stem cells clusters of cancer tissues (**Figure 7E**) while generally expressed low in the cell clusters of paraneoplastic tissue (**Figure 7F**). This suggests that the increased expression of RARRES3 is consistent with the trend of increased immune cell infiltration in tumor tissues.

### 3.7 Correlation of TP53 mutations with the immune landscape

**Figure 8:**
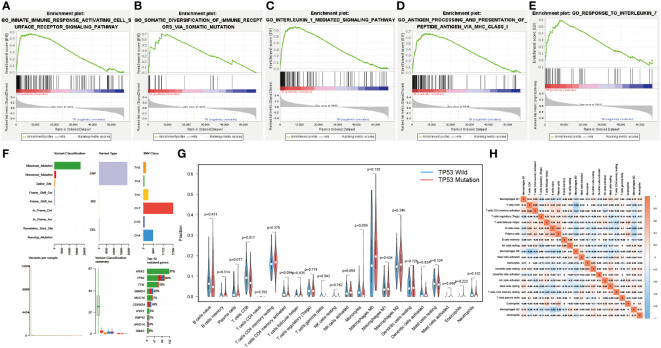
Correlation of TP53 mutations with the immune landscape. **(A-E)** TP53 mutation group is significantly enriched in five immune-related biological processes in GSEA. **(F)** Overall mutation type and mutation percentage analysis of TP53 in TCGA-PAAD cohort. **(G)** Different immune cell infiltration in wild and mutant groups. **(H)** Correlation matrix of all 22 immune cell proportions.

RARRES3 (alternate name PLAAT4) and TNFSF10 are hub genes of TiME-score. As a co-upstream regulatory gene of RARRES3 and TNFSF10, TP53 mutation has attracted our attention for its relevance to TiME. Although the correlation between TP53 mutations and OS in PAAD patients has been well substantiated (13–15), the effect of TP53 mutation on PAAD-specific TiME has not been thoroughly investigated. We performed GSEA analysis of PAAD samples from the TP53 wild group (n=62) and mutation group (n=81) using gene expression and clinical information from TCGA. Five immune-related biological processes were selected based on the results: innate immune response activating cell surface receptor signaling pathway (NES=1.805, FDR=0.034, SIZE=115), somatic diversification of immune receptors via somatic mutation (NES=1.741, FDR=0.049, SIZE=18), interleukin 1 mediated signaling pathway (NES=1.983, FDR =0.029, SIZE=99), antigen processing and presentation of peptide antigen via MHC class I (NES=1.891, FDR=0.028, SIZE=97), and response to interleukin 7 (NES=1.876, FDR=0.025, SIZE=39) (**Figures 8A-E**). The results showed that the TP53 mutation was significantly enriched in immune-related biological processes. However, the TP53 wild group had no gene sets significantly enriched at FDR < 25%. Moreover, the mutation data analysis of the TCGA-PAAD cohort showed that missense mutations, SNPs, and C>T accounted for a large proportion of TP53 mutations, which with a high mutation rate of 63% (**Figure 8F**). Further analysis of the differences in the TiME landscape between the TP53 wild and mutant groups revealed the presence of more pronounced immunosuppression in the mutant group compared to the wild group (**Figure 8G**). The proportion of tumor-infiltrating immune cells in different subpopulations was weakly to moderately correlated (**Figure 8H**).

### 3.8 Prognostic value of hub gene RARRES3

**Figure 9:**
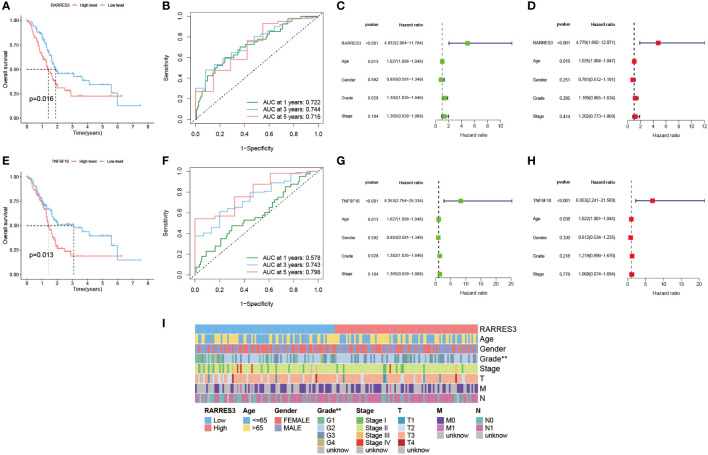
Prognostic value of hub gene RARRES3. **(A)** Survival analysis of RARRES3 high and low expression groups. **(B)** ROC curves to assess the categorical performance of RARRES3. **(C)** Univariate independent prognostic analysis of RARRES3. **(D)** Multivariate independent prognostic analysis of RARRES3. **(E-H)** The same method was used to perform survival analysis and independent prognostic analysis of TNFSF10. **(I)** Clinical factor analysis of high and low RARRES3 expression groups.

In this study, the prognostic value of RARRES3 was further explored. Considering that RARRES3 and TNFSF10 are both downstream genes of TP53, and TNFSF10 is a validated prognostic risk factor for PAAD, we used TNFSF10 as a comparator to evaluate the prognostic value of RARRES3. Survival analysis showed that survival time was significantly lower in the RARRES3 high-expression group than in the low-expression group in the TCGA-PAAD cohort (P=0.016) (**Figure 9A**). The ROC curves also demonstrated good sensitivity in predicting the prognosis (AUC at 1 year: 0.722, AUC at 3 years: 0.744, AUC at 5 years: 0.716) (**Figure 9B**). Furthermore, independent prognostic analysis of univariate and multifactorial showed that RARRES3 had significantly better independent prognostic value than other relevant clinical factors after integration (P<0.001) (**Figures 9C, D**). Using the same method to analyze TNFSF10, TNFSF10 exhibited a similar positive prognostic value to RARRES3 (**Figures 9E-H**). This also indirectly verified the prognostic ability of RARRES3. The clinical information of the high and low RARRES3 expression groups was additionally analyzed as a whole, and we found a significant correlation between RARRES3 and clinical grading (**Figure 9I**). Samples with high RARRES3 expression had a higher level of clinical staging than those with low expression, with a higher proportion of Grade 3 and 4. This suggests that high RARRES3 expression corresponds to worse clinical staging.

### 3.9 Positive regulation of RARRES3 and macrophages M1

**Figure 10:**
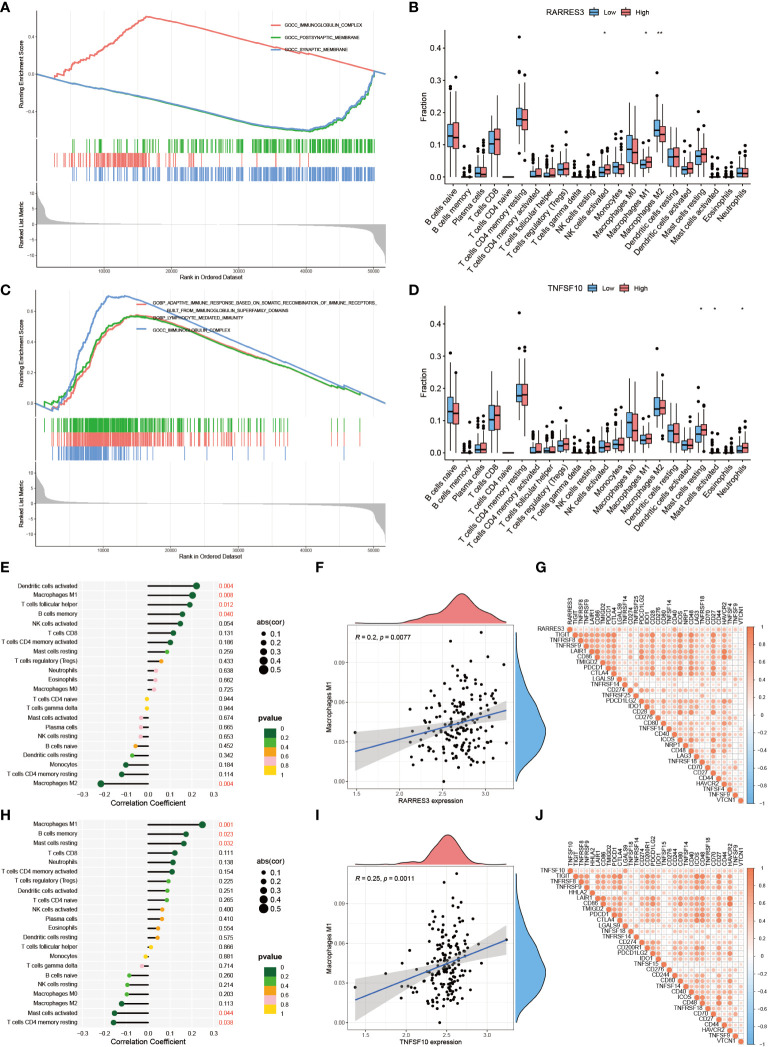
Positive regulation of RARRES3 and Macrophages M1. **(A)** Top three biological enrichment processes of RARRES3. **(B)** Immune cell infiltration in high and low RARRES3 expression groups (“**”: P<0.01, “*”: P<0.05). **(C, D)** Analysis of TNFSF10 by the same method **(E)** Immune cell correlation analysis of RARRES3. **(F)** Correlation between RARRES3 expression and Macrophages M1 **(G)** Immunological check site correlation analysis of RARRES3 **(H–J)** Immunological correlation analysis of TNFSF10 using the same method.

GSEA analysis was applied to RARRES3 and TNFSF10 separately, taking the top three ranked biological enrichment processes (**Figures 10A, C**). It was found that the immune-related component IMMUNOGLOBULIN COMPLEX was significantly enriched in the high expression group of both genes. Next, immune cell infiltration was analyzed for RARRES3 and TNFSF10 (**Figures 10B, D**). In particular, the high infiltration characteristics of Macrophages M1 in the high expression group attracted our additional attention. Further analysis of the correlation between all immune cells and RARRES3 expression was performed (**Figure 10E**). The results showed that Macrophages M1 was positively correlated with RARRES3 expression (R=0.2, P=0.0077) (**Figure 10F**). Additionally, RARRES3 was positively correlated with all immune checkpoint-related genes (**Figure 10G**). Then, TNFSF10 was analyzed in the same way, and the positive correlation of TNFSF10 expression levels with Macrophages M1 was significantly similar to RARRES3 (**Figures 10H-J**). The positive correlation of RARRES3 with pro-inflammatory Macrophages M1 and the negative correlation with anti-inflammatory Macrophages M2 (R=-0.004) also led to our speculation of the pro-inflammatory TiME in the RARRES3 high expression group.

### 3.10 Assessment of immune landscape and immunotherapy response in high- and low-risk groups

**Figure 11:**
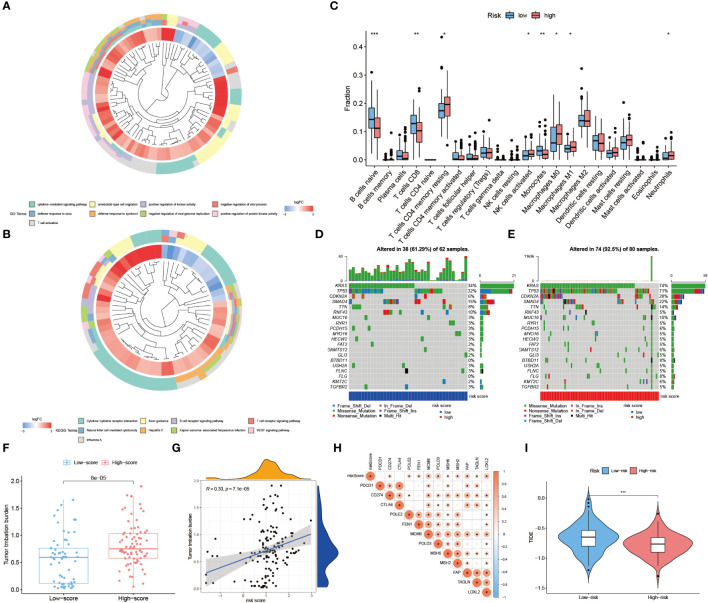
Assessment of immune landscape and immunotherapy response in high- and low-risk groups. **(A, B)** GO biological function enrichment analysis and KEGG pathway enrichment analysis of hub genes. **(C)** Differences of 22 immune cell infiltration abundance in high and low risk groups. **(D, E)** TMB scores for each mutated sample in high and low risk groups. **(F)** Quantitative analysis of TMB in high and low risk groups. **(G)** TiME-score and TMB correlation analysis. **(H)** Correlation analysis between TiME-score and immune check points. **(I)** Differences in TIDE scores between high and low risk groups. (“***”: P<0.001, “**”: P<0.01, “*”: P<0.05).

The enrichment analysis of GO and KEGG showed that the hub genes of TiME-score were mainly enriched in the biological processes of immune activation, immune defense, and immune system-related diseases (**Figures 11A, B**, **Supplementary Table 4**). A further assessment of the infiltration of immune cells in the high- and low-risk groups was performed (**Figure 11C**). B-cell naive phase, T-cell CD8+, T-cell CD4+ memory phase, monocytes, and macrophage M0 were found to be negatively correlated with TiME-score; while macrophage M1, activated NK cells were positively correlated with TiME-score. We also calculated TMB scores for each mutant sample in the TCGA-PAAD cohort (**Figures 11D, E**). The results showed that the high-risk group had a broader TMB distribution, and the proportion of TP53 mutations was significantly higher in the high-risk group (71%) than in the low-risk group (32%). Furthermore, TMB quantitative analysis confirmed statistically significant differences in TMB distribution between high and low risk groups (Wilcoxon test, p=8e-05; **Figure 11F**). A positive correlation between TiME-score and TMB was also confirmed (R=0.33, p=7.1e-05; **Figure 11G**). To further clarify the relationship between TiME-score and immunotherapy response, we also explored the correlation between immune check sites and TiME-score (**Figure 11H**). Correlation analysis showed that CD274, FAP, FEN1, LOXL2, MCM6, MSH2, MSH6, POLD3, POLE2, and TAGLN were all positively correlated with TiME-score. In addition, the TIDE scores of patients in the high-risk and low-risk groups indicated that patients with low-risk were more likely to experience immune escape and patients with high-risk were more likely to benefit from immune checkpoint inhibitor (ICI) therapy (**Figure 11I**).

### 3.11 Validation of hub genes in immune-, targeted- and chemo-therapy mice PAAD model

**Figure 12:**
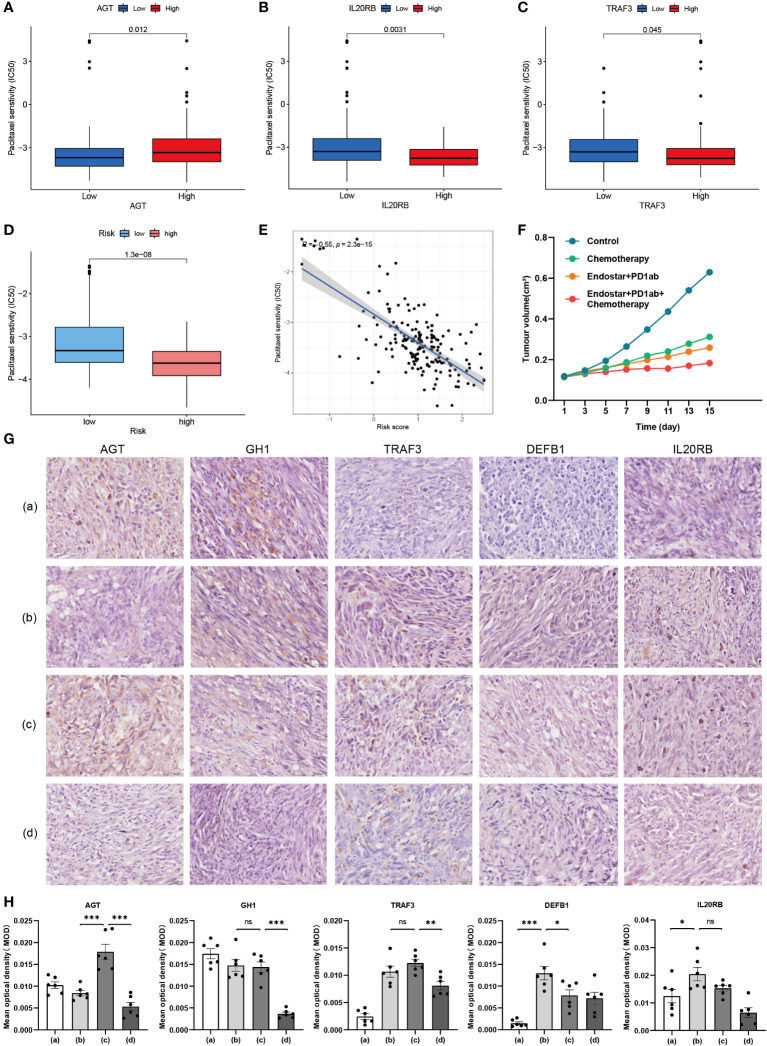
Validation of hub genes in immune-, targeted- and chemo-therapy mice PAAD model. **(A–C)** Differences in IC50 of Paclitaxel between high and low expression groups of AGT, IL20RB and TRAF3. **(D)** Differences in IC50 of Paclitaxel between high and low risk groups of TiME-score. **(E)** IC50 correlation analysis of TiME-score and Paclitaxel. **(F)** Tumor growth curves of 4 different mice PAAD treatment cohorts. **(G)** Immunohistochemistry of 5 hub genes in 4 different treatment cohorts (a: Model Control Group; b: Chemotherapy Group; c: Recombinant Human Endostar Injection + PD-1 Monoclonal Antibody Group; d: Recombinant Human Endostar Injection + PD-1 Monoclonal Antibody + Chemotherapy Group) **(H)** Mean optical density (MOD) analysis of 5 hub genes in 4 different treatment mice cohorts (“***”: P<0.001, “**”: P<0.01, “*”: P<0.05).

Clinical treatment of PAAD with single drug is much less effective than combination therapy (16). Nano-albumin Paclitaxel in combination with Gemcitabine is the first chemotherapy choice for non-surgical PAAD patients (17). Currently, immunotherapy, represented by PD-1 monoclonal antibody, is not as effective in combination with chemotherapy alone or with other molecules (18). In contrast, some clinical therapeutic efficacy has been achieved by combining recombinant human vascular endothelial inhibitor with chemotherapy (19). Therefore, we further explored the intergroup differences in IC50 of these drugs. We selected GH1, for which immunohistochemical differences were not significant, TRAF3, for which immunohistochemical trend were opposite to transcriptomic and proteomic trends, DEFB1, for which proteomic and transcriptomic trends did not match, IL20RB, for which neither immunohistochemical nor proteomic was retrieved, and the TiME-score itself for separate analysis. AGTs with consistent transcriptomic, immunohistochemical, and proteomic trends were included in the analysis as reference. Among them, Paclitaxel had a lower IC50 in the AGT low expression group (**Figure 12A**), indicating that AGT low expressers were more sensitive to Paclitaxel. The IC50 was lower in the IL20RB (**Figure 12B**) and TRAF3 (**Figure 12C**) high expression groups, indicating that IL20RB and TRAF3 high expressers were more sensitive to Paclitaxel. For the TiME-score (**Figures 12D, E**), the IC50 of Paclitaxel was lower in the high-risk group (R = -0.55, p = 2.3e-15), indicating that those with higher scores were more sensitive to Paclitaxel. Unfortunately, there were no statistically significant group differences in GH1 and DEFB1 to Paclitaxel. There were no significant between-group differences for Gemcitabine. Recombinant human vascular endothelial inhibitors were not included in the pRRophetic package used for prediction. However, considering the value of their combination therapy mentioned above, we still included the combination regimen of Nano-albumin Paclitaxel and Gemcitabine as the chemotherapy group and included recombinant human vascular endothelial inhibitor in the targeted combination chemotherapy group. Combining the above research advances, 4 different regimen treatment cohorts of model control group, chemotherapy group (Nano-albumin Paclitaxel + Gemcitabine), recombinant human vascular endothelial inhibitor + PD-1 monoclonal antibody group and recombinant human vascular endothelial inhibitor + PD-1 monoclonal antibody + chemotherapy group were included in this study. Volume recordings of the tumors for 15 consecutive days (**Figure 12F**) showed that the tumor growth volume and rate in the recombinant human vascular endothelial inhibitor + PD-1 monoclonal antibody + chemotherapy group were significantly smaller than those in the remaining 3 groups. This suggests the value of combining immunotherapy with targeted therapy and chemotherapy for tumor suppression. Next, we performed immunohistochemical validation of GH1, TRAF3, DEFB1, and IL20RB, which are controversial above, in mice PAAD model (**Figures 12G, H**). AGT, for which transcriptomic, immunohistochemical, and proteomic trends were consistent, was also included as a reference for the feasibility and accuracy of this validation approach. The results showed that AGT with a negative coefficient (Coef. = -0.0661) was expressed at a lower level in the Nano-albumin Paclitaxel group than non-Nano-albumin Paclitaxel group. DEFB1 (Coef. = 0.0159) and IL20RB (Coef. = 0.0479) with positive coefficients had the higher expression in the Nano-albumin Paclitaxel involved chemotherapy group. This is consistent with the lower IC50 of Paclitaxel in the AGT low expression group and IL20RB high expression group. Considering the positive correlation coefficient of the hub gene in TiME-score, the expression of the gene was positively correlated with the score and those with high score were more sensitive to Nano-albumin Paclitaxel. The experiments for DEFB1 were also consistent with the predicted results. The intergroup differences for GH1 (Coef.= -3.2585) and TRAF3 (Coef.= -0.3324), although the P value was not smaller than 0.05, expression differences were consistent with the predicted trend. Moreover, considering immune cell infiltration was more pronounced in the low-risk group, DEFB1 and IL20RB with positive coefficients were significantly less expressed in the immunotherapy cohort than in the non-immunotherapy cohort. AGT with a negative coefficient had higher expression in the immunotherapy cohort. In other words, the immunotherapy cohort corresponded to a lower TiME-score, and the low-risk group had better immunotherapy outcomes and a better prognosis. Collectively, TiME-score is potentially valuable as an immune-related prognostic model for the prediction of individualized PAAD anticancer drug sensitivity.

## 4 Discussion

Highly immunosuppressed TiME is a key barrier to immunotherapy in PAAD. Immune cell fractions are the most common indicators of TiME levels (20). The extracellular matrix, the vascular system, and cancer-associated fibroblasts, which account for up to 80-90% of the total, provide a difficult “natural barrier” for cancer cells to escape from immune and drug tracking (21). Furthermore, signals released by the cancer cells themselves, such as aberrant activation of the pro-oncogene K-Ras and overexpression of MYC protein genes, also play crucial roles in generating a deeply immunosuppressive environment for PAAD (22, 23). To further reflect the TiME profile and its association with the prognosis of PAAD patients, this study selected the 14 genes with the most prognostic value and constructed a TiME-score by combining immune-related IRGs, TFs, and clinical data. Among them, the prognostic value of CXCL9, ERAP2, MET, PLAU, and TYK2 in PAAD has been fully studied (24–28). GH1 and IL20RB have also been shown to be associated with the proliferation and development of PAAD (29, 30). Although the impact of DEFB1 in PAAD has not been proven, its specific deletion as the only innate immune gene with long-term balancing selection and heterozygote advantage promotes the development of kidney and prostate cancers (31, 32). In turn, the knockdown gene expression profile of TRAF3, which is an inhibitor of the NFκB pathway, is associated with better survival and better immune checkpoint blockade response in primary cancer-treated patients having immune checkpoint blockade therapy (33).

TP53 is a co-upstream regulator gene shared by RARRES3 and TNFSF10, which are the hub genes of the TiME-score. Hsu, T.H., et al. suggested that TP53 function in cell proliferation and Wnt/β-linked protein signaling in breast cancer was significantly associated with the induction of RARRES3 (34). TNFSF10 is a tumor risk factor that mediates TP53-dependent cell death (35). Qu, Y., et al. suggested that in hepatocellular carcinoma TP53 regulates TNFSF10-induced apoptosis by modulating the function of the TNFSF10 receptor (36). On the other hand, TP53, as the second mutated factor in the PAAD genome, plays a non-negligible role in promoting PAAD invasion, PD-L1 kinetics, and immune evasion (15).TP53 mutations can not only inhibit innate immune responses through tumor-associated macrophages and neutrophil surface toll-like receptors but also reduce ULBP1- and ULBP2-mediated anti-tumor activity of NK cells and affect the number of T cell infiltrates through dendritic cells (37, 38). In this study, the TiME landscape of TP53 mutant and wild groups were further analyzed. The results showed that the proportion of CD8+ T cells was significantly lower in the TP53 mutant group than in the wild group and that TP53 mutations were weakly to moderately correlated with changes in the proportion of tumor-infiltrating immune cells and different subpopulations. Taking into account that TP53 mutations can lead to lipase H overexpression, which in turn reduces the expansion of CD8+ T cells and Th1 cells (39), the low density of CD8+ cells in PAAD tumor centers could suggest a tumorigenic effect and poor prognosis of immunosuppressive TiME (40). In addition, CD8+ T cell infiltration is negatively correlated with the expression of fibrosis-related genes, and TP53 mutations can also cause PAAD-TiME suppression by enhancing the ability of extracellular matrix deposition (41).

Subsequently, RARRES3, a hub gene of the TiME-score, attracted our additional attention as a retinol-induced class II tumor suppressor gene whose downregulation often leads to metastasis of cancer cells (42). In breast cancer, RARRES3 downregulation can lead to tumor cell adhesion involved in metastasis initiation, loss of RARRES3 phospholipase A1/A2 activity can lead to impaired tumor cell differentiation, and RARRES3 has the potential to act as an endogenous inhibitor of immunoproteasome expression (43, 44). RARRES3 can also participate in the regulation of hepatocarcinogenesis through the miR-1/G9a/RARRES3 axis, and in the regulation of epithelial-mesenchymal transition in colorectal cancer through the inhibition of MTDH (45, 46). In the field of PAAD, RARRES3 has also been included as a prognostic indicator to predict PAAD development and metastasis (47), but has not been validated for its immunoprognostic value. Through independent prognostic value analysis as well as immune landscape analysis of RARRES3, we found that the high RARRES3 expression group had a more pronounced immune correlation and a worse prognosis than the low expression group. It is interesting to note that in further analysis of the immune cell infiltration landscape of the RARRES3 high expression group, we found that RARRES3 expression levels showed a significant positive correlation with pro-inflammatory M1-type macrophages and a significant negative correlation with anti-inflammatory M2-type macrophages. However, in the majority of tumors TiME, M1 often plays the role of a protection factor, while M2 mainly plays the role of promoting tumor growth, invasion, and metastasis. We speculate that this may be related to the specificity of TiME in PAAD. On the one hand, Zhang, M., et al. suggested that PAAD tumor cells can selectively induce glycolytic methylation and OXPHOS gene down-regulation in M1-like macrophages through direct cell-cell contact with M1-like macrophages, leading to a suppressed glycolytic state in M1-like macrophages, which is not present in M2-like macrophages (48). Upon interaction with PAAD tumor cells, M1-like macrophages can then be phenotypically reprogrammed to M2-like macrophages and thus acquire pro-oncogenic capacity. On the other hand, Chang, Y.-T., et al. suggested that Small extracellular vesicles (sEVs)-Ezrin in PAAD-TiME could regulate macrophage polarization, tumor-associated macrophages reprogrammed to M2 phenotype, and promote PDAC metastasis (49). Furthermore, Ma, X., et al. suggested that overexpression of REG4 secreted by PAAD tumor cells could promote macrophage polarization to M2 through at least partial activation of ERK1/2 and CREB, and modify TiME to promote the growth and metastasis of PAAD (50). Summarizing, combined with our analysis of M1-type macrophages in PAAD TiME, we speculate that the positive correlation between poor prognosis and M1-type macrophages in the RARRES3 high expression group can be explained by reprogramming of M1. And blocking this M1 to M2 re-editing emerges as a new potential way to improve the outcome of PAAD immunotherapy.

Besides, previous studies have shown that TNFSF10 can induce autophagy through the MAPK8 activation pathway of TRAF2 and RIPK1, which in turn blunts the apoptosis of cancer cells (51). In the immune monitoring of tumor cells, TNFSF10 can also function to control inflammation by inducing apoptosis of macrophages and neutrophils (52). TNFSF10 and its corresponding death receptor signaling can also regulate cancer metastasis (53). Considering the shared upstream gene TP53 and the apparent homology with RARRES3, TNFSF10 was used as a well-referenced gene to validate the immune-related prognostic value of RARRES3 in this study.

PAAD monotherapy is much less clinically effective than combination therapy (16). Immunotherapy, including monoclonal antibodies, immune checkpoint inhibitors, pericytes, vaccines, and other agents that enhance the antitumor response or reverse the immunosuppressive function of regulatory immune cells in the microenvironment, has made great progress in the treatment of cancer, including PAAD, in recent decades (54). However, immunotherapy represented by PD-1 monoclonal antibody is currently not effective in PAAD either use alone (55) or in combination with chemotherapy only (18). Albumin-conjugated nanocarriers of Paclitaxel have improved the efficacy of the first-line pancreatic cancer drug Gemcitabine by inhibiting the tumor stroma and suppressing the expression of the gemcitabine-inactivating enzyme cytidine deaminase (56). In PAAD patients without surgical indication and in good physical condition, Nano-albumin Paclitaxel (Abraxane^®^) in combination with Gemcitabine is the first choice of chemotherapy (17). Nano-albumin Paclitaxel plus Gemcitabine significantly improves overall survival, progression-free survival and remission rates in PAAD patients (57). And the combination of recombinant human vascular endothelial inhibitor (Endostar^®^) with chemotherapy has been achieving some clinical therapeutic results (19). The Recombinant Human Vascular Endothelial Inhibitor is an important drug to address angiogenesis in TiME. Recombinant Human Vascular Endothelial Inhibitor in combination with PD-1 monoclonal antibody can have a significant impact on tumor growth by improving TiME and activating autophagy (58). Recombinant Human Vascular Endothelial Inhibitor, PD-1 monoclonal antibody together with chemotherapy has demonstrated favorable near-term efficacy and safety in the first-line treatment of driver gene-negative advanced non-squamous non-small cell lung cancer (59). But so far, no studies of Recombinant Human Vascular Endothelial Inhibitor combined with PD-1 inhibitors and conventional chemotherapy in PAAD have been reported. Hence, considering the value of combination therapy, 4 different treatment cohorts of mice PAAD model were designed in this study: chemotherapy group (Abraxane + Gemcitabine), Recombinant Human Vascular Endothelial Inhibitor + PD-1 monoclonal antibody group, Recombinant Human Vascular Endothelial Inhibitor + PD-1 monoclonal antibody + chemotherapy group, and model control group. While exploring the best treatment modality for PAAD patients, the expression of the hub genes of TiME-score was verified. Immunohistochemical results showed differential trends in the expression of AGT, DEFB1, GH1, IL20RB, and TRAF3 between groups consistent with the results of drug sensitivity analysis and immunoscape analysis in high and low risk groups. The prognostic value of the immune-related TiME-score and the value of guiding individualized pharmacological use were further validated.

There are also some drawbacks to this study. First, the hub gene RARRES3 was not validated in the mice PAAD model because only antibodies applicable to humans were identified. We speculate that this is associated with the non-expression of the RARRES3 gene and proteins in mice, and will be further verified in subsequent studies in human PAAD immune clinical trials. Moreover, the present study is mainly based on public databases and mice models which deserve further validation in a prospective clinical cohort receiving immunotherapy. And future integrated multi-omics analysis would be helpful to compensate for the current limitation on transcriptional, mutational, and clinical data.

## 5 Conclusion

In conclusion, the TiME-Score based on RARRES3 has promising potential for predicting the prognosis of PAAD patients and correlates closely with the efficacy of immunotherapy. The excellent ability of this comprehensive signature to predict prognosis provides an appropriate individualized immunotherapy strategy for PAAD patients.

## Data availability statement

The original contributions presented in the study are included in the article/**Supplementary Material**. Further inquiries can be directed to the corresponding authors.

## Ethics statement

The animal study was approved by Animal Ethics Committee of Jiangsu University. The study was conducted in accordance with the local legislation and institutional requirements.

## Author contributions

YF and XW provided direction and guidance throughout the preparation of this manuscript. YS wrote and edited the manuscript. RH completed the section of molecular sub types identification. CM, LY and RH collected related papers and made significant revisions to the manuscript. All authors read and approved the final manuscript.
