# Supplementary material for: Construction and validation of a RARRES3-based prognostic signature related to the specific immune microenvironment of pancreatic cancer
Source: Front Oncol. 2024 Feb 5;14:1246308. doi: 10.3389/fonc.2024.1246308 (PMC10876156; doi:10.3389/fonc.2024.1246308)
Supplement: Supplementary file 1 [file DataSheet_1.zip › SupplementaryMaterial/Captions for supplementary materials.docx]

**Construction and validation of a RARRES3-based prognostic signature related to the specific immune microenvironment of pancreatic cancer**

1. **Supplementary Figure Captions**

**Supplementary Figure S1. Basis of determining the number of clusters.**

Clustering of the TCGA-PAAD cohort when the number of clusters k is taken as 2 (**A**), 4 (**B**), 5 (**C**), 6 (**D**), 7 (**E**), 8 (**F**) and 9 (**G**). (**H**) Consistent cumulative Distribution Function (CDF) plots for different number of clusters (**I**) Delta Area Plot for different number of clusters.

**Supplementary Figure S2. Differential expression of the remaining 10 hub genes between PAAD and normal tissues.**

Expression pattern of CXCL9 (**A**), DEFB1 (**B**), GH1 (**C**), IL20RB (**D**), LMBR1L (**E**), PLAU (**F**), RARRES3 (**G**), TNFSF10 (**H**), TRAF3 (**I**), TYK2 (**J**) in normal and PAAD samples from the PAAD and GTEx databases.

**Supplementary Figure S3. Proteomic differences of hub genes between PAAD and normal tissues.**

Proteomic differences of AGT (**A**), DEFB1 (**B**), ERAP2 (**C**), MET (**D**), PLAU (**E**), TNFSF10 (**F**), TRAF3 (**G**), TYK2 (**H**) and their Statistical parameters (**I**) between normal and PAAD samples from the CPTAC database.

**Supplementary Figure S4. Expression and distribution of the hub genes of TiME-score in PAAD single cell samples.**

(**A**) All cells in 3 PAAD single-cell samples were clearly divided into 10 cell clusters. (**B**) Accuracy verification of each cell cluster annotation. (**C**) Differences in expression and distribution of all 14 hub genes of TiME-score in these 10 cell clusters.

**Supplementary Figure S5. Expression and distribution of the hub genes of TiME-score in PAAD para-neoplastic normal tissue single-cell samples.**

(**A**) All cells in the 1 PAAD para-neoplastic normal tissue single-cell sample were clearly divided into 13 cell clusters. (**B**) Accuracy verification of each cell cluster annotation. (**C**) Except for GH1, which was not expressed in this sample, expression and distribution differences of all the TiME-score hub genes in these 13 cell clusters.

1. **Supplementary Table Captions**

**Supplementary Table S1. Dosing regimen of 4 groups of mice models.**

**Supplementary Table S2. Immunohistochemistry antibody information.**

**Supplementary Table S3. 152 Immune Genes Associated with PAAD Prognosis.**

**Supplementary Table S4. Top 9 Biological Processes of GO and KEGG Enrichment Analysis in The High and Low Risk Groups.**
