# Supplementary figures and images for: Construction and validation of a RARRES3-based prognostic signature related to the specific immune microenvironment of pancreatic cancer
Source: Front Oncol. 2024 Feb 5;14:1246308. doi: 10.3389/fonc.2024.1246308 (PMC10876156; doi:10.3389/fonc.2024.1246308)

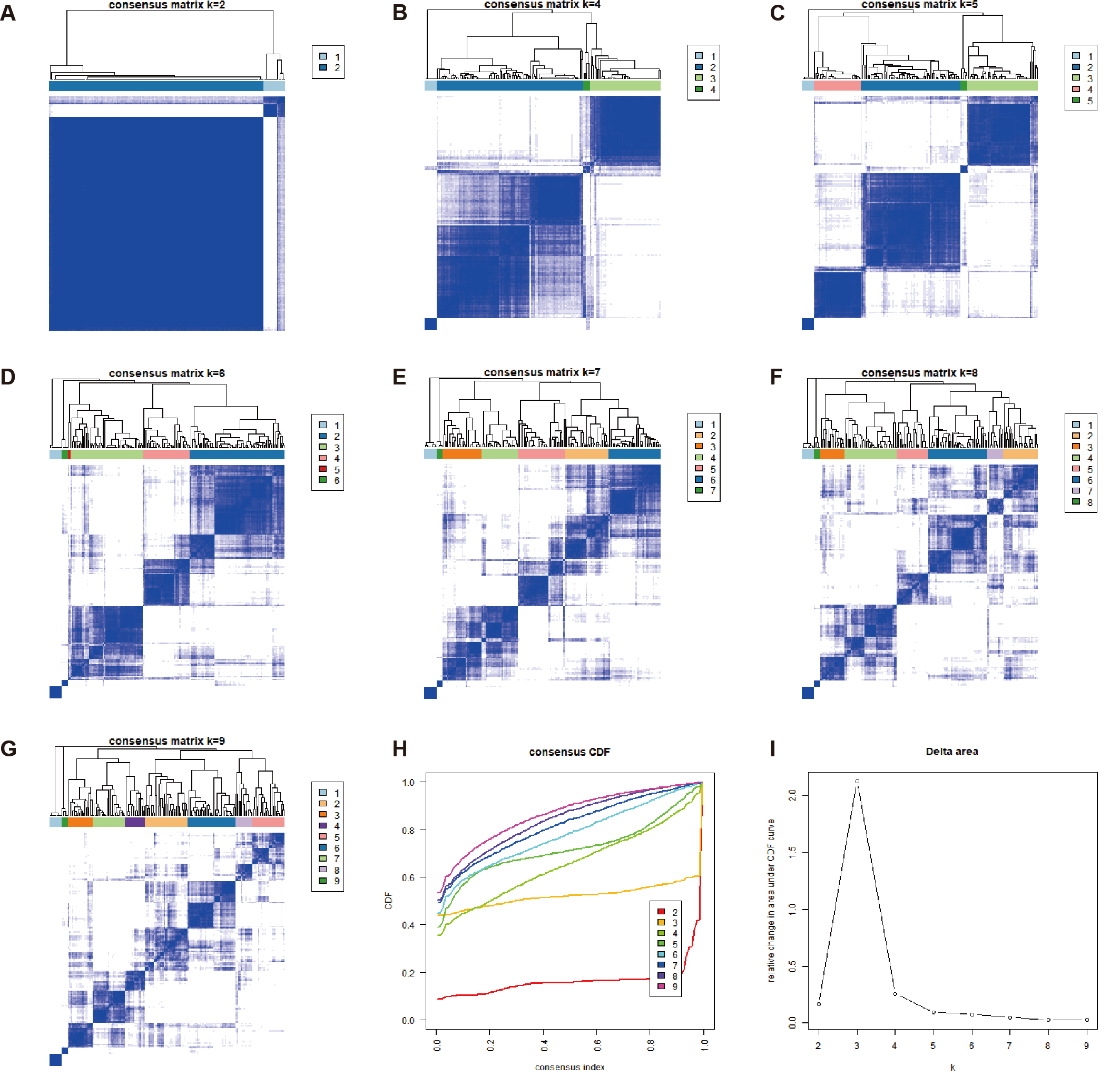

Supplement: Supplementary file 1 [file DataSheet_1.zip › SupplementaryMaterial/Figure_S1_Basis of determining the number of clusters.tif]

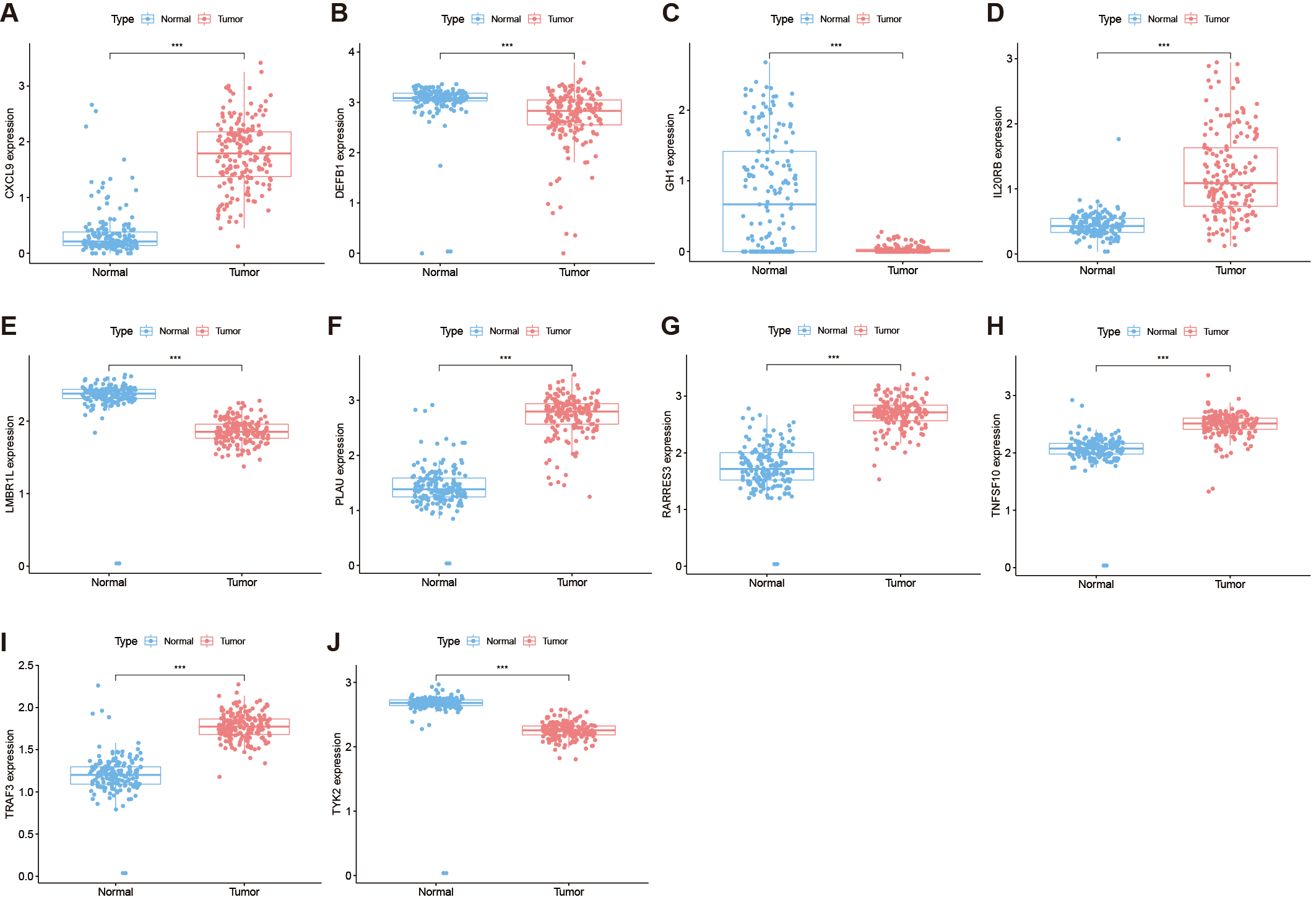

Supplement: Supplementary file 1 [file DataSheet_1.zip › SupplementaryMaterial/Figure_S2_Differential expression of the remaining 10 hub genes between PAAD and normal tissues.tif]

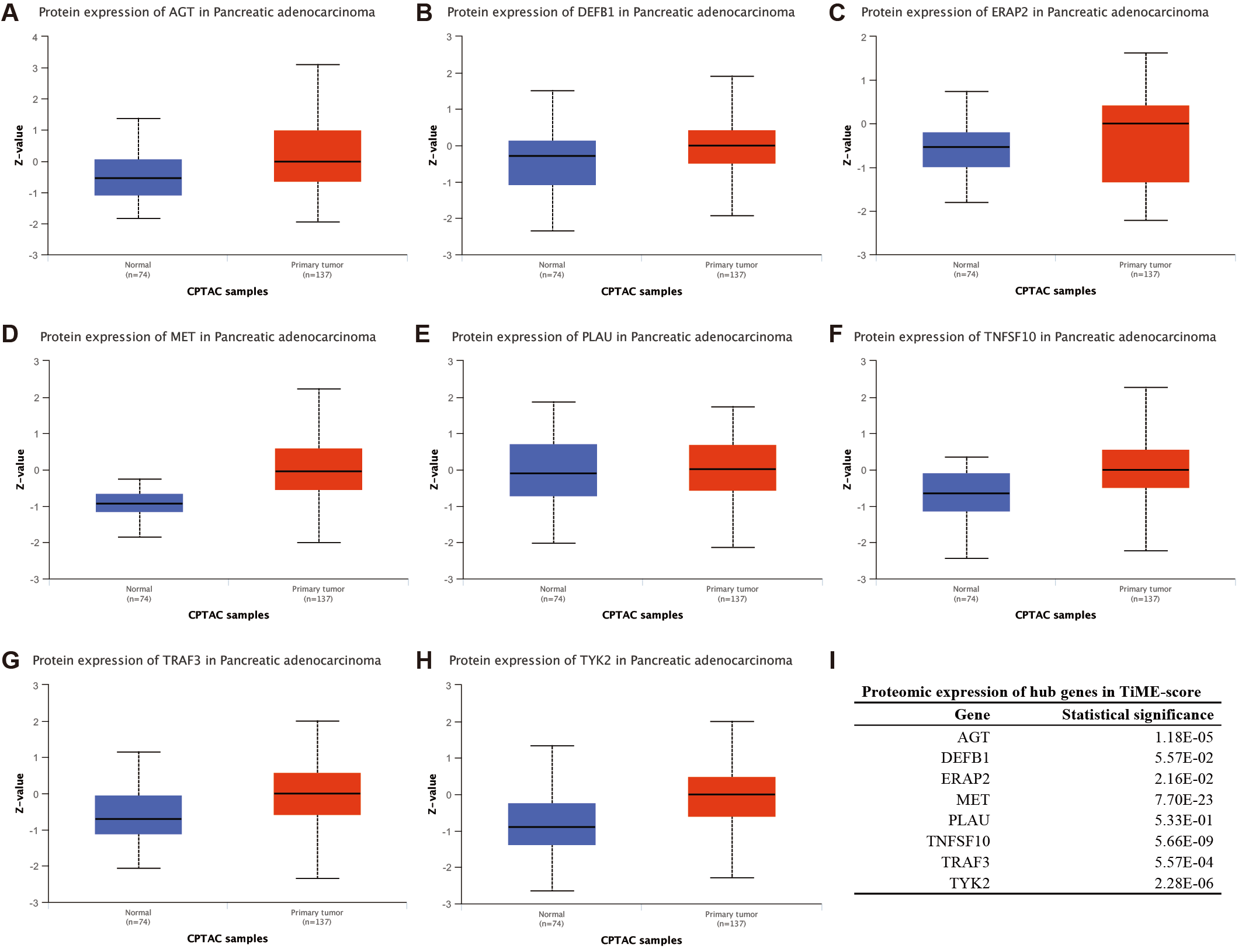

Supplement: Supplementary file 1 [file DataSheet_1.zip › SupplementaryMaterial/Figure_S3_Proteomic differences of hub genes between PAAD and normal tissues.tif]

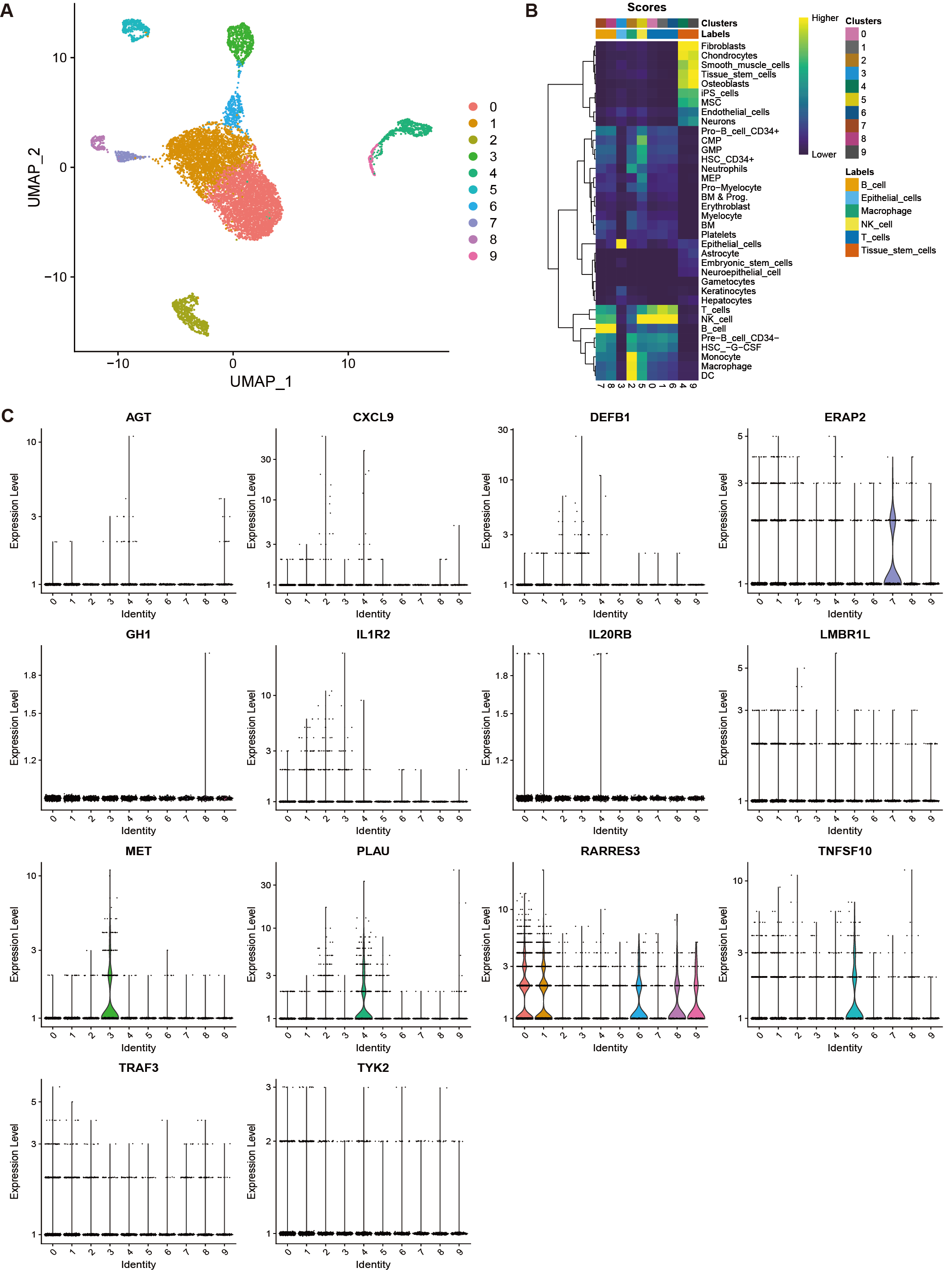

Supplement: Supplementary file 1 [file DataSheet_1.zip › SupplementaryMaterial/Figure_S4_Expression and distribution of the hub genes of TiME-score in PAAD single cell samples.tif]

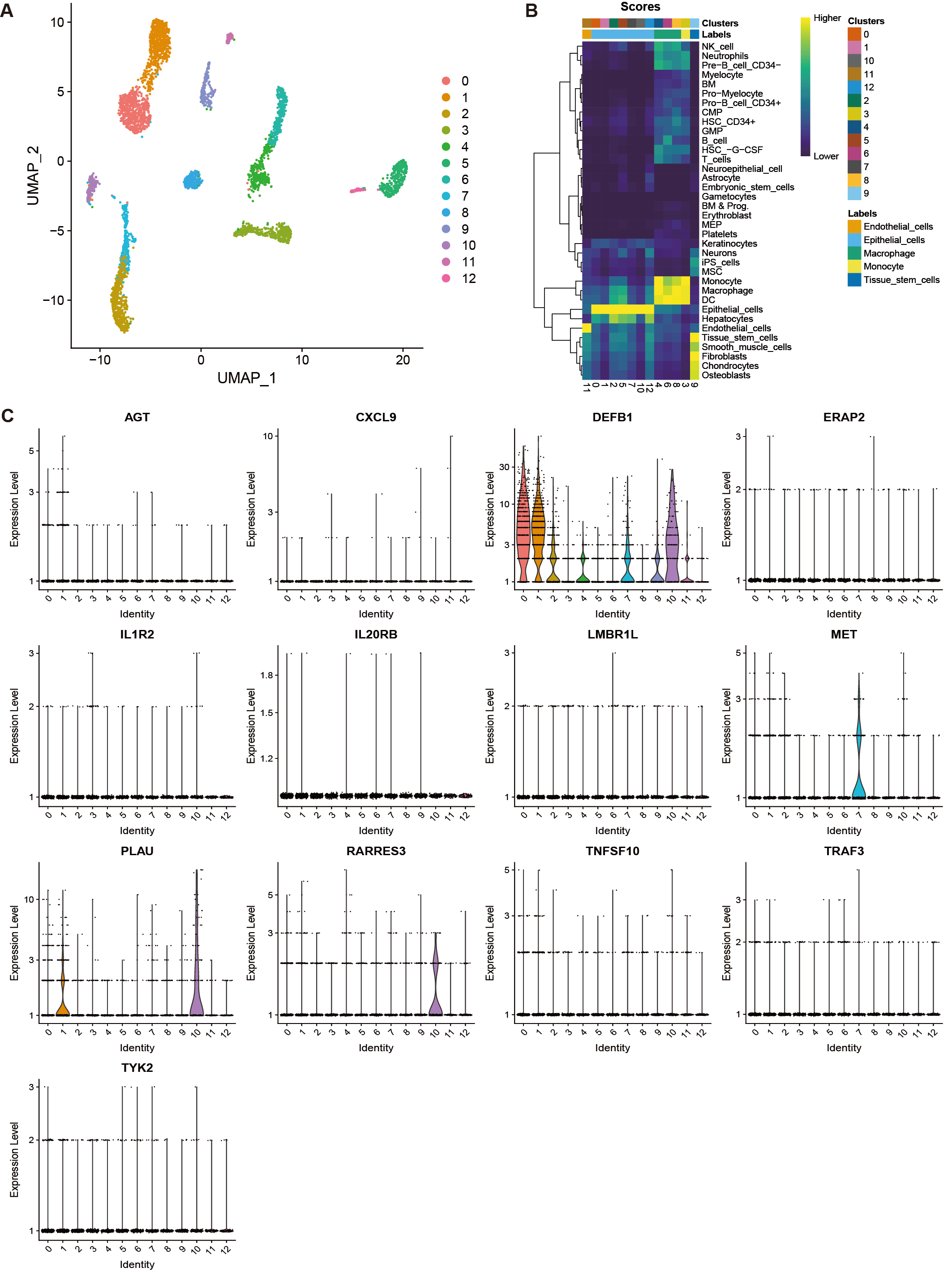

Supplement: Supplementary file 1 [file DataSheet_1.zip › SupplementaryMaterial/Figure_S5_Expression and distribution of the hub genes of TiME-score in PAAD para-neoplastic normal tissue single-cell samples.tif]
